# Supplementary material for: Root transcriptomic responses of grafted grapevines to heterogeneous nitrogen availability depend on rootstock genotype
Source: J Exp Bot. 2017 Jul 11;68(15):4339–55. doi: 10.1093/jxb/erx224 (PMC5854021; doi:10.1093/jxb/erx224)
Supplement: Supplementary_Figures [file erx224_suppl_supplementary_figures.pdf]

Title:

Root transcriptomic responses of grafted grapevine to heterogeneous N availability depend on rootstock genotype

Authors:

Noé Cochetel, Frédéric Escudié, Sarah Jane Cookson, Zhanwu Dai, Philippe Vivin, Pierre-François Bert, Mindy Stephania Muñoz, Serge Delrot, Christophe Klopp, Nathalie Ollat, Virginie Lauvergeat

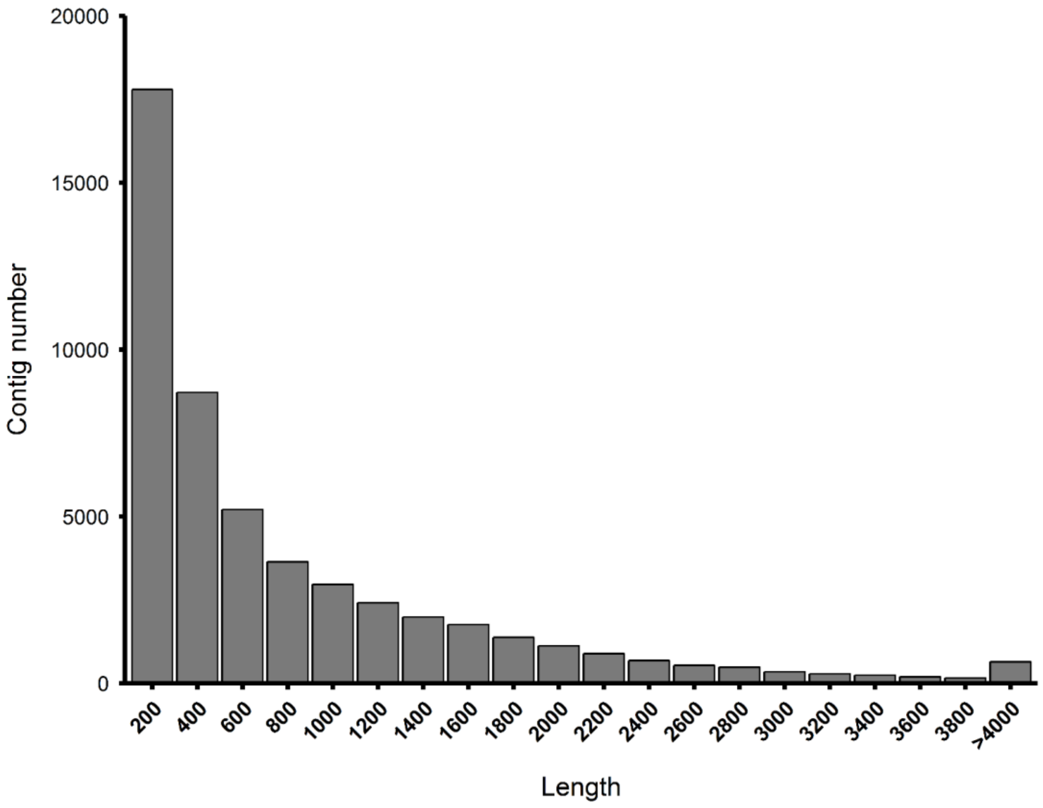

Supplementary Figure S1. *Length distribution of* de novo *assembled contigs*
Values are comprised between 201 and 15,529 bp; and contigs from 201 to 599 bp represent half of the whole transcriptome.

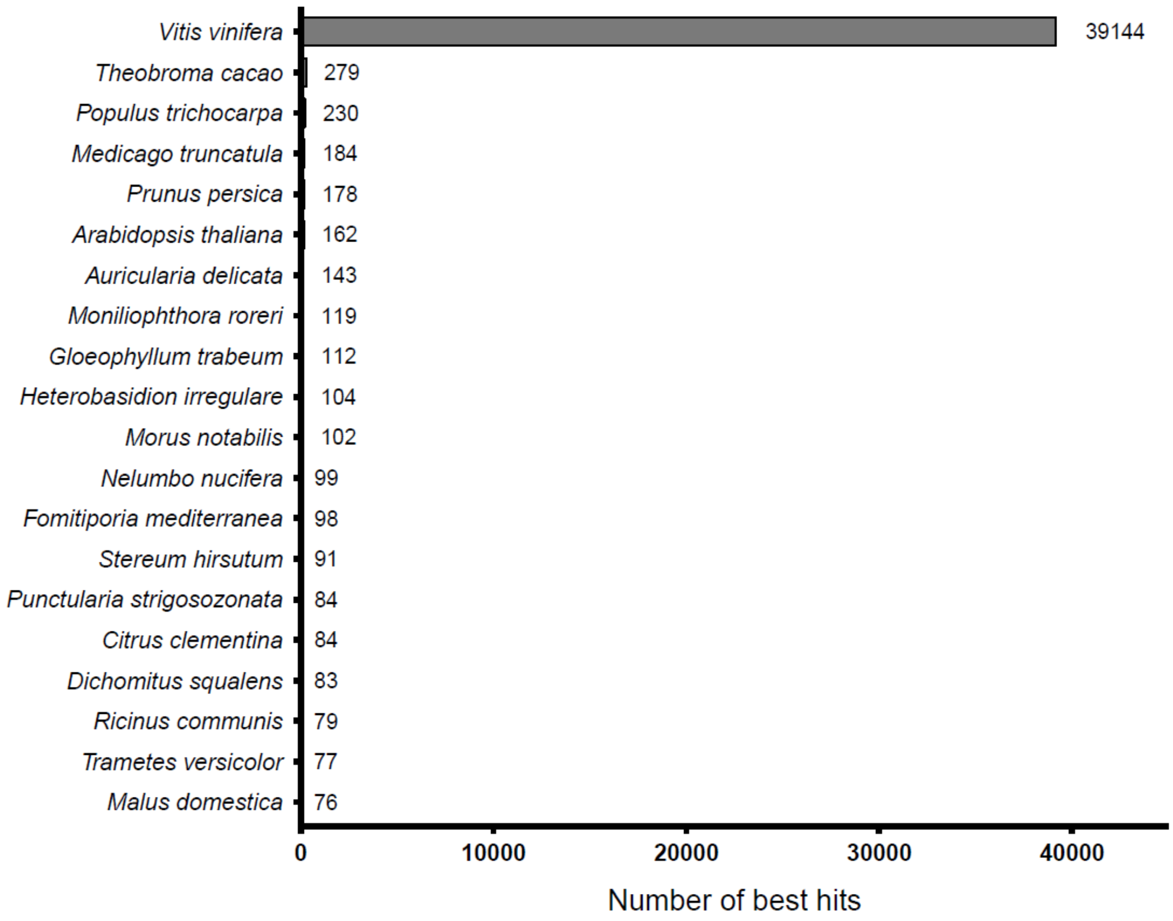

Supplementary Figure S2. *Top-hit species distribution of the merged final transcriptome*
The 20 most represented species in the transcriptome annotation are presented.

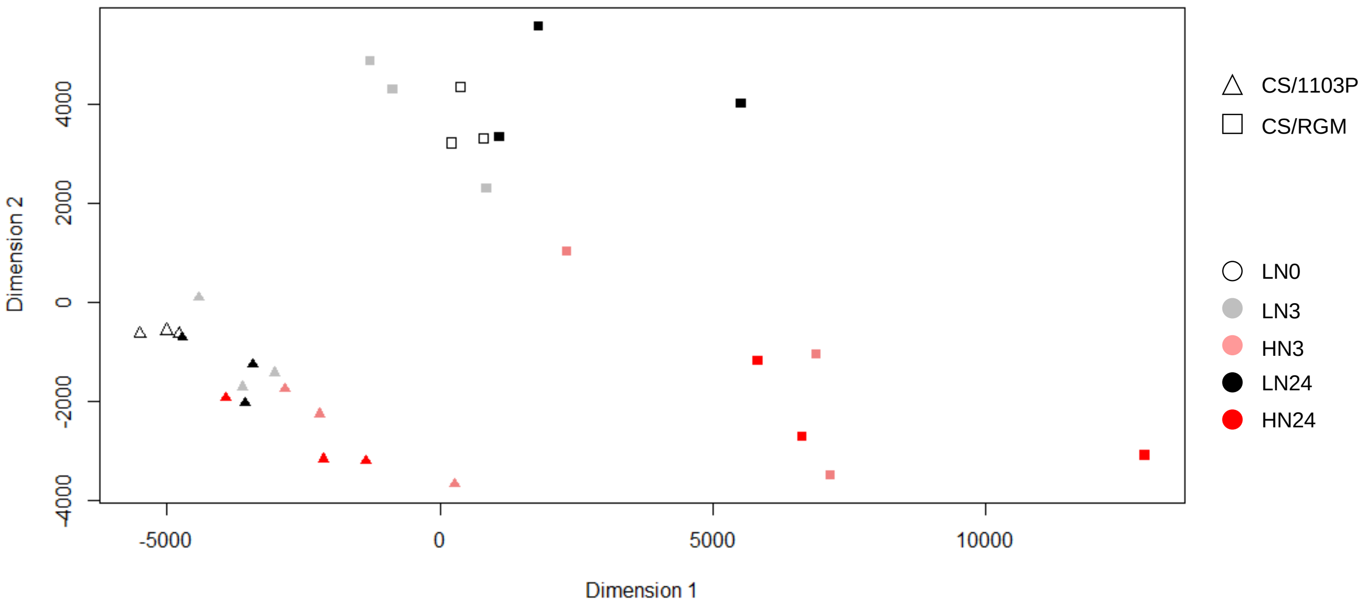

Supplementary Figure S3. *Multi-Dimensional Scaling plot of counts data from the different RNAseq samples*

Each condition is represented by three biological replicates with different colors for each time and N treatment. The combination CS/1103P is indicated using triangles and CS/RGM using squares.

A

Bin	BinName	Contingency	Pvalue	Adj.Pvalue (Bonf.)
17	hormone metabolism	29 913 183 50125	5.56E-17	5.95E-15
17.5	hormone metabolism.ethylene	24 150 188 50888	1.75E-29	1.87E-27
17.5.2	hormone metabolism.ethylene.signal transduction	24 56 188 50982	2.18E-38	2.33E-36
12	N-metabolism	6 49 206 50989	1.14E-07	1.22E-05
12.1	N-metabolism.nitrate metabolism	3 1 209 51037	2.78E-07	2.98E-05
12.1.2	N-metabolism.nitrate metabolism.nitrite reductase	2 0 210 51038	1.70E-05	1.82E-03
7	OPP	9 50 203 50988	3.14E-12	3.36E-10
7.3	OPP.electron transfer	6 6 206 51032	4.22E-12	4.52E-10
7.1.1	OPP.oxidative PP.G6PD	3 12 209 51026	3.06E-05	3.28E-03
27.3.5	RNA.regulation of transcription.ARR	7 23 205 51015	3.52E-11	3.77E-09
27.3.20	RNA.regulation of transcription.G2-like transcription factor family. GARP	4 49 208 50989	7.11E-05	7.61E-03
19	tetrapyrrole synthesis	5 61 207 50977	8.41E-06	9.00E-04
19.3	tetrapyrrole synthesis.uroporphyrin-III C-methyltransferase	5 0 207 51038	1.16E-12	1.24E-10
34.4	transport.nitrate	8 14 204 51024	2.28E-14	2.44E-12

B

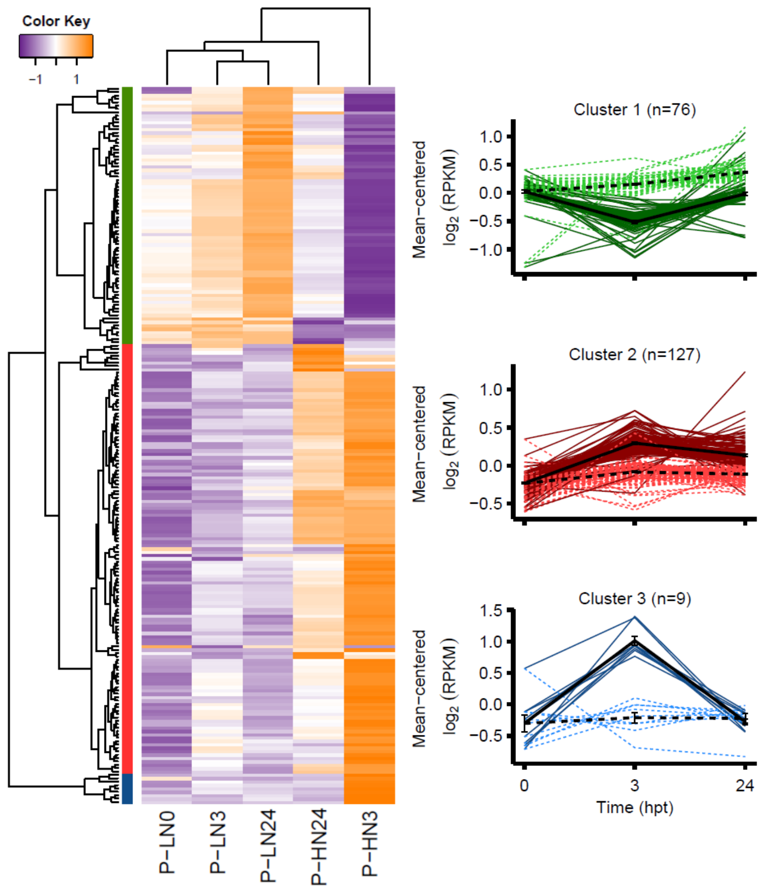

Supplementary Figure S4. *The few number of DEGs responding to N availability in CS/1103P involves mainly N-related genes*

A) Enrichment analysis of the DEGs from CS/1103P (n=212). Contingency gives the number of genes (i) in the BIN in the input list, (ii) in the background, (iii) not in the BIN in input list, and (iv) not in the background. *P*-values were adjusted with Bonferroni. Values were filtered with an adjusted *P*-value threshold <0.01 and an enrichment >1. B) Hierarchical clustering of the transcripts and the different conditions (*i.e.* Treatment x Harvesting time) in a heatmap presenting the expression pattern of each DEGs (raws) within the different samples (columns). The harvesting time 0 hpt was excluded during the hierarchical clustering process. Expression values are RPKM log_2-transformed with up-regulation to down-regulation varying from orange to purple colour. Transcript clusters were extracted using the gene hierarchical clustering tree. The x-axis represents the harvesting time (in hpt), y-axis represents the mean-centered RPKM log_2-transformed values. HN and LN conditions were drawn in dark coloured solid and light coloured dashed lines, respectively. Mean ± SE was represented in black colour.

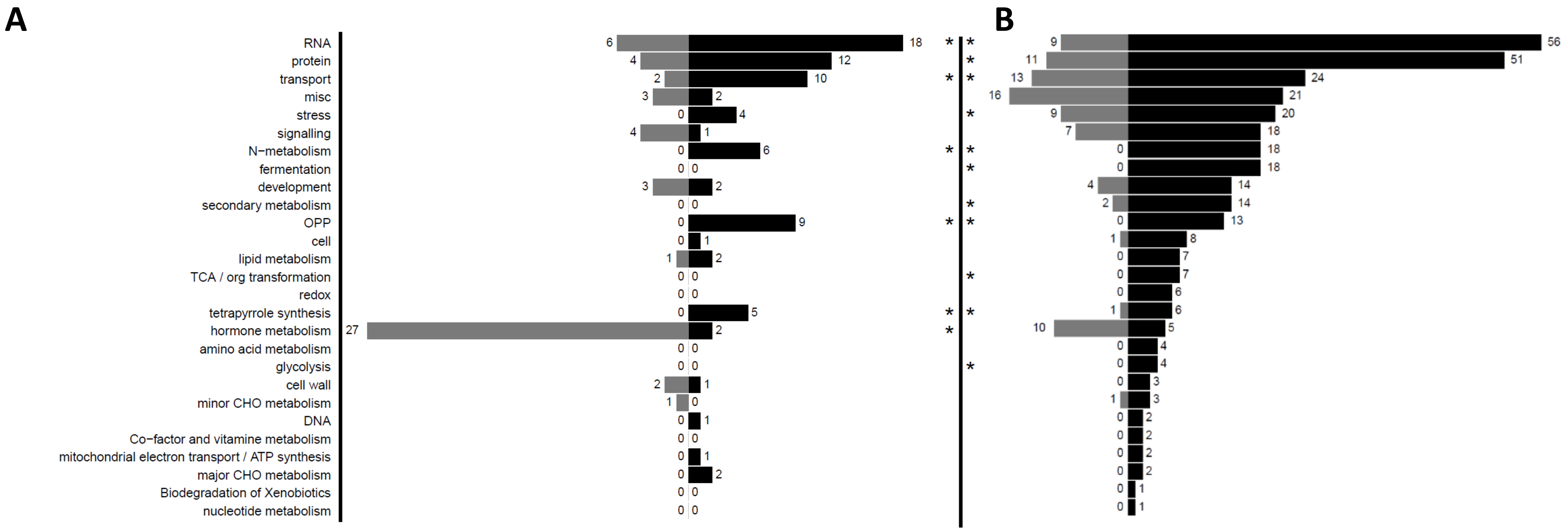

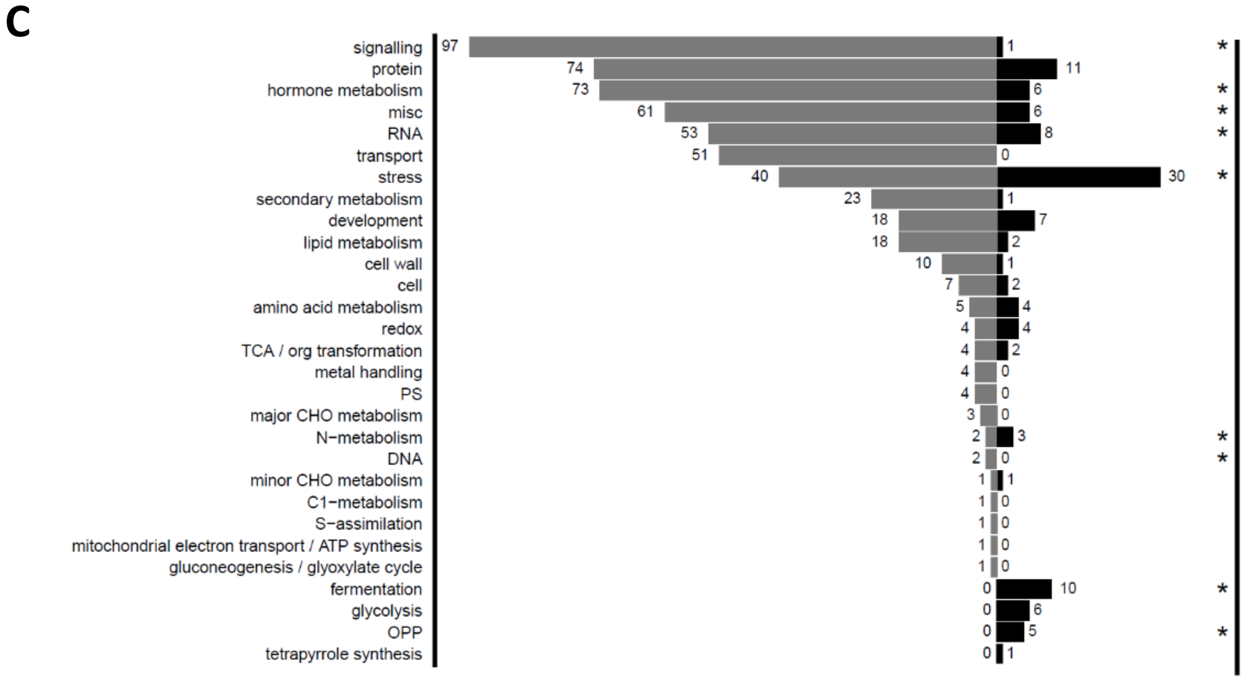

Supplementary Figure S5. *Functional categories distribution within DEGs*
Histograms show the functional categories attributed to DEGs from contrasts between HN and LN-irrigated roots at 3 hpt in CS/1103P (A), CS/RGM (B) and at 24 hpt in CS/RGM only (C). Asterisks represent significative enrichment resulting of a mefisto analysis with an adjusted *P*-value (Bonferroni) < 0.01. Up and down-regulated genes were represented in black and grey colors, respectively.

Sample	Name	Raw reads	Cleaned reads	Filtered (%)
PFB-56_20_S2	P-LN0.1	44022375	43367351	1.49
PFB-57_6_S1	P-LN0.2	38152919	37876907	0.72
PFB-58_12_S1	P-LN0.3	33703044	33450549	0.75
PFB-29_22_S1	P-LN3.1	42601769	41923422	1.59
PFB-31_11_S2	P-LN3.2	32059193	31565788	1.54
PFB-36_25_S2	P-LN3.3	36641969	36073755	1.55
PFB-30_10_S1	P-HN3.1	42260609	41587058	1.59
PFB-35_15_S2	P-HN3.2	34314981	33797028	1.51
PFB-37_7_S1	P-HN3.3	38166051	37489125	1.77
PFB-41_1_S1	P-LN24.1	40729211	40006233	1.78
PFB-43_23_S2	P-LN24.2	40366903	39696276	1.66
PFB-48_19_S2	P-LN24.3	39746879	38920801	2.08
PFB-42_13_S1	P-HN24.1	40898469	40175340	1.77
PFB-47_12_S2	P-HN24.2	42133688	41323911	1.92
PFB-49_9_S1	P-HN24.3	37070521	36641692	1.16
PFB-53_2_S1	R-LN0.1	36326877	35866299	1.27
PFB-54_16_S1	R-LN0.2	47255815	46613390	1.36
PFB-55_18_S2	R-LN0.3	44216349	43660318	1.26
PFB-29_22_S1	R-LN3.1	42601769	41923422	1.59
PFB-31_11_S2	R-LN3.2	32059193	31565788	1.54
PFB-36_25_S2	R-LN3.3	36641969	36073755	1.55
PFB-33_3_S1	R-HN3.1	44614433	43981469	1.42
PFB-38_4_S1	R-HN3.2	38706051	38067161	1.65
PFB-40_11_S2	R-HN3.3	41161470	40505828	1.59
PFB-44_7_S2	R-LN24.1	39427555	38666683	1.93
PFB-46_6_S1	R-LN24.2	44895875	43964087	2.08
PFB-51_14_S2	R-LN24.3	35281333	34863971	1.18
PFB-45_5_S1	R-HN24.1	41343213	40285076	2.56
PFB-50_2_S1	R-HN24.2	52795478	52172562	1.18
PFB-52_16_S2	R-HN24.3	44618538	44012991	1.36

Supplementary Table S1. *Read number before (raw) and after trimming (cleaned)*
The sample names contain a letter depending on each rootstock genotype; P: CS/1103P, R: CS/RGM, followed by HN/LN; HN: High Nitrate solution, LN: Low Nitrate solution; the harvesting time and the number of the replicate are separated by a dot.

Assembly	Nb seq	N50	L50	Lg sum	Lg min	Lg lower quartile	Lg mean	Lg median	Lg upper quartile	Lg max	% re-mapping	Nb prot aligned (80%/80%)	Nb contig with prot(s)
oases_rpkm01	51250	1481	9820	47 519 774	201	328	927.22	576	1218	15529	89.73	17950	15001
oases_rpkm03	44050	1594	8469	43 041 069	201	324	977.10	604	1330	15529	89.33	17600	14555
oases_rpkm10	20853	1943	4510	26 571 035	202	378	1274.21	984	1799	15529	83.96	13722	10636
trinity_rpkm01	71430	1253	12263	53 429 994	201	269	748.00	416	895	15529	88.66	17458	15086
trinity_rpkm03	55962	1440	9711	46 137 723	201	270	824.45	445	1072	15529	88.09	17068	14484
trinity_rpkm10	21431	1869	4469	25 480 402	201	337	1188.95	868	1697	15529	81.84	13094	10293

Supplementary Table S2. *Summary table of the* de novo *assemblies*
Each assembler, Oases and Trinity, was used with three different RPKM parameters 1; 3 and 10. Nb seq is the total contigs number obtained, N50 is defined as the largest entity E such that at least half of the total size of the entities is contained in entities larger than E and L50 is the number of scaffolds that accounts for more than 50% of the genome assembly. Data on contig length (Lg) include the sum of all contig lengths (Lg sum), the minimum length (Lg min), lower quartile, mean, median, upper quartile and the maximum length (Lg max). Reads were mapped back to the different assemblies using BWA (% re-mapping) (Li and Durbin, 2009). Protein alignment was performed by BLAT (Kent, 2002) and counted if the protein coverage is upper than 80% and/or with an identity upper than 80%. The number of contigs with protein(s) was determined in the same way by counting contigs with one or more protein associated after the filtering process (coverage and/or identity of 80%).

Accession number	Gene name	Function	Forward primer (5’-3’)	Reverse primer (5’-3’)
VIT_05s0020g02480	*GS*	Glutamine synthetase	CTGTCTCCCTTGCTCCCTCTC	GGAGTCACTGGAAATGGGTTGAAC
VIT_18s0001g03910	*NR*	Nitrate reductase	GCTGGTGTTCTGAATGATGGAG	AAGACGACGCTAGGAGAGAAG
VIT_03s0063g00370	*NIR*	Nitrite reductase	CTTGCCGAGGAAGTGGAATGTG	GCATGTACGCCAGATCATTGATGT
VIT_02s0154g00260	*NPF6.3*	NRT1/PTR Family 6.3	CAGTTCTTATTGGTGGGTGC	CCCTAATGAAAGTGTGCTCAAA
VIT_06s0061g00320	*NRT2.4a*	Nitrate transporter 2.4a	CCTCCCACCTTCAAAGGA	CATGGGATGGTGTAGAGTTGG
VIT_08s0040g01500	*NRT2.4b*	Nitrate transporter 2.4b	AGGTGGGAACTTCGGATC	TCTTTGGAGGGCGGTAGC
VIT_17s0000g09470	*NRT3*	Nitrate transporter 3	CTTGACATTGCTGCCGTTTG	GGAAACAGTCATAGTGGCTAACTT
VIT_17s0000g10430	*GAPDH*	Glyceraldehyde-3-Phosphate Dehydrogenase	CCACAGACTTCATCGGTGACA	TTCTCGTTGAGGGCTATTCCA
VIT_12s0035g01130	*EF1γ*	Elongation factor 1 gamma	CAAGAGAAACCATCCCTAGCTG	TCAATCTGTCTAGGAAAGGAAG

Supplementary Table S3. *List of the primers used for qPCR experiments*
